# Role of Diffusion Tensor Imaging Parameters in the Characterization and Differentiation of Infiltrating and Non-Infiltrating Spinal Cord Tumors: Preliminary Study

**DOI:** 10.1007/s00062-019-00851-8

**Published:** 2019-11-21

**Authors:** Edyta Maj, Barbara Szemplińska, Wojciech Szeszkowski, Marek Prokopienko, Andrzej Cieszanowski, Andrzej Marchel, Olgierd Rowiński

**Affiliations:** 1 2nd Department of Clinical Radiology, Medical University of Warsaw, ul. Zwirki i Wigury 61, 02-091 Warsaw, Poland; 2 Department of Neurosurgery, Institute of Psychiatry and Neurology, ul. Sobieskiego 9, 02-957 Warsaw, Poland; 3 1st Department of Radiology, Maria Skłodowska-Curie Memorial Cancer Centre and Institute of Oncology, ul. Roentgena 5, 02-781 Warsaw, Poland; 4 Department of Neurosurgery, Medical University of Warsaw, ul. Zwirki i Wigury 61, 02-091 Warsaw, Poland

**Keywords:** Spinal cord neoplasms, Astrocytoma, Ependymoma, Magnetic resonance imaging, Diffusion tensor imaging

## Abstract

**Background and Purpose:**

Recent attempts to utilize diffusion tensor imaging (DTI) to identify the extent of microinfiltration of a tumor in the brain have been successful. It was therefore speculated that this technique could also be useful in the spinal cord. The aim of this study was to differentiate between infiltrating and noninfiltrating intramedullary spinal tumors using DTI-derived metrics.

**Material and Methods:**

The study group consisted of 6 patients with infiltrating and 12 with noninfiltrating spinal cord tumors. Conventional magnetic resonance imaging (MRI) with gadolinium administration was performed followed by DTI. Fractional anisotropy (FA), diffusivity (TRACE) and apparent diffusion coefficient (ADC) were measured in the enhancing tumor mass, peritumoral margins, peritumoral edema and normal appearing spinal cord. The results were compared using non-parametric Mann–Whitney *U* test with statistical significance *p* < 0.05.

**Results:**

In peritumoral margins the FA values were significantly higher in the noninfiltrating compared to the infiltrating tumors (*p* < 0.007), whereas TRACE values were significantly lower (*p* < 0.017). The results were similar in peritumoral edema. The FA values in the tumor mass showed no significant differences between the two groups while TRACE showed a statistically significant difference (*p* < 0.003). There was no statistical difference in any parameters in normal appearing spinal cord.

**Conclusion:**

Quantitative analysis of DTI parameters of spinal cord tissue surroundings spinal masses can be useful for differentiation between infiltrating and non-infiltrating intramedullary spinal tumors.

## Introduction

Diffusion tensor imaging (DTI) and diffusion tensor tractography are emerging magnetic resonance imaging (MRI) techniques, which depict course and integrity of white matter tracts by tracking the diffusion of water molecules [1]. Recent studies evaluated the use of DTI parameters to define the extent of tumor microinfiltration beyond the apparent borders on T2-weighted and contrast-enhanced images in the brain [2]; however, few have evaluated its use in the diagnosis of spinal cord lesions. The lack of such publications is attributed to a combination of technical difficulties [3] of using this method in the spinal cord, and to the low incidence of spinal cord lesions. Primary spinal cord tumors in particular are 10 times less common than their cranial counterparts [4] and represent only 2–4% of all central nervous system (CNS) neoplasms [5]. The most common intramedullary spinal cord tumors are ependymomas and astrocytic tumors, followed by hemangioblastomas [6, 7]. While the morphological MRI of a hemangioblastoma does not usually present a diagnostic challenge, the differentiation between an astrocytoma and an ependymoma often does [8].

Surgery is indicated in essentially all spinal cord tumors. Resective surgery can usually be accomplished in spinal ependymomas by separating the tumor from the spinal cord. If complete separation can be achieved, no further treatment is required. By contrast, spinal cord gliomas infiltrate the myelin and consequently surgery is almost always incomplete. Involved-field radiotherapy is most often administered after partial resection [5]. This is why differentiating infiltrating and non-infiltrating spinal cord tumors is essential in the determination of optimal patient management strategies. Research suggests that infiltrating tumors (e.g astrocytomas) have a higher water molecular diffusion heterogeneity and a higher degree of tissue nonintegrity around the tumor mass as compared to noninfiltrating tumors (e.g ependymomas). This study aimed to differentiate infiltrating from noninfiltrating intramedullary spinal tumors using DTI-derived metrics from the enhancing parts of tumor and peritumoral regions.

## Material and Methods

### Subjects

Data acquired over a 7-year period (2008–2016) were retrospectively analyzed. A total of 18 patients who underwent surgery and in whom primary spinal cord tumor was histologically confirmed were enrolled in the study. The mean age of all patients at the time of diagnosis was 34.8 years (ranging from 20 to 49 years) and for sex there was a slight female predominance (11:7). Detailed information on all 18 patients is provided in Table 1. One patient with a histologically confirmed spinal cord metastasis and four with a hemangioblastoma were excluded from the analysis, as well as one patient with an acute demyelinating process and one with cord ischemia diagnosed during further clinical follow-up.

**Table 1:** Characteristics and diagnoses of patients included in the study

Case	Age (years)	Sex	Diagnosis	WHO grading	Location in the spinal cord
1	28	M	Astrocytoma	2	Med. obl. – C2
2	30	M	Ependymoma	2	C2 – C5
3	27	F	Primitive neuroectodermal tumor	4	C2 – C5
4	49	F	Ependymoma	2	Med. obl
5	39	F	Ependymoma	2	Med. obl. – C3
6	24	F	Glioblastoma	4	Med. obl. – C1
7	33	F	Astrocytoma	2	C3 – Th1
8	38	M	Ependymoma	2	C3 – C7
9	46	M	Ependymoma	2	C6 – Th3
10	34	F	Ependymoma	2	C3 – C4
11	25	F	Ependymoma	2	C1 – C5
12	57	F	Ependymoma	2	C7-Th1
13	29	M	Ependymoma	2	C5 – Th1
14	29	M	Ependymoma	2	Th9 – Th10
15	44	F	Ependymoma	2	C6 – Th1
16	32	F	Ependymoma	2	Th9 – Th11
17	20	F	Diffuse glioma	3	Med. obl
18	42	M	Glioblastoma	4	Th3 – Th6

*M* man, *F* female, *C* cervical spine, *Th* thoracic spine, *Med.obl.* medulla oblongata

Based on the histological results subjects were divided into two groups: those with infiltrating (IT; 6 patients), and those with non-infiltrating tumors (NIT; 12 patients). Analysis of MRI scans and patient classification was performed by EM (board certified neuroradiologist, 10 years experience in image analysis) and BS (general radiologist in training).

### MRI Examinations

Routine MRI scans were performed using a 1.5‑T unit (Siemens Magnetom Avanto SQ Engine TIM 76 × 32, Erlangen, Germany). Written informed consent was obtained from all patients before MRI examination. The study protocol was approved by the institution’s ethics review board.

The following images were obtained for anatomical and diagnostic purposes: a sagittal T2 TSE (TR = 3000 ms; TE = 102 ms; 15 slices 3 mm thick); an axial T2 heavily T2* weighted 2D spoiled gradient echo multi-echo sequence (multi-echo data image combination-MEDIC) (TR = 502 ms; TE = 17 ms; matrix 256 × 256; slice thickness 3 mm) and a sagittal T1 3D MPRAGE (Magnetization Prepared RApid Gradient Echo) (TR = 2100 ms; TE = 2.9 ms; isotropic resolution 1 mm^3^), both before and after the injection of a gadolinium chelate at a standard dose of 0.1 mmol/kg body weight.

The following parameters were used for acquiring the DTI in the axial plane: TE = 86 ms; TR = 3100 ms; iPAT = 2; NEX 1; FOV 230; matrix 128 × 128; voxel size 1.9 × 1.9 × 5 mm, slice thickness 5 mm; 30 gradient directions; b values = 0 s/m^2^;1000 s/mm^2^. The DTI sequence covered the area from the midbrain to below the tumor mass (TM). The examination was performed in one slice group. The average DTI sequence duration was 10 min and 48 s.

### Analysis

The trace and anisotropy are two common measures of the diffusion tensor [9]. The trace of the tensor (TRACE) or sum of the diagonal elements of the diffusion coefficient, measures the magnitude of diffusion and is rotationally invariant. The mean diffusivity, often called the apparent diffusion coefficient (ADC), is the trace divided by three, equivalent to the average of the eigenvalues. The degree to which the diffusivities are a function of the diffusion-weighted encoding direction is represented by measures of diffusion anisotropy [9]. The most widely used invariant measure of anisotropy is fractional anisotropy (FA) [10]. For calculation of FA, TRACE and ADC maps the imaging software Syngo.via (Siemens, Germany) was used. Regions of interest (ROIs) of a fixed small size (0.15 cm^2^, 4 pixels) were manually defined on axial colored FA maps and then reported to ADC and TRACE maps to avoid partial volume effects, magnetic susceptibility effects and motion artifacts. Both authors (BS and EM) reached a consensus as to the definition of the ROIs. The radiologists were blinded to the tumor types at the time of performing the measurements.

**Fig. 1:**
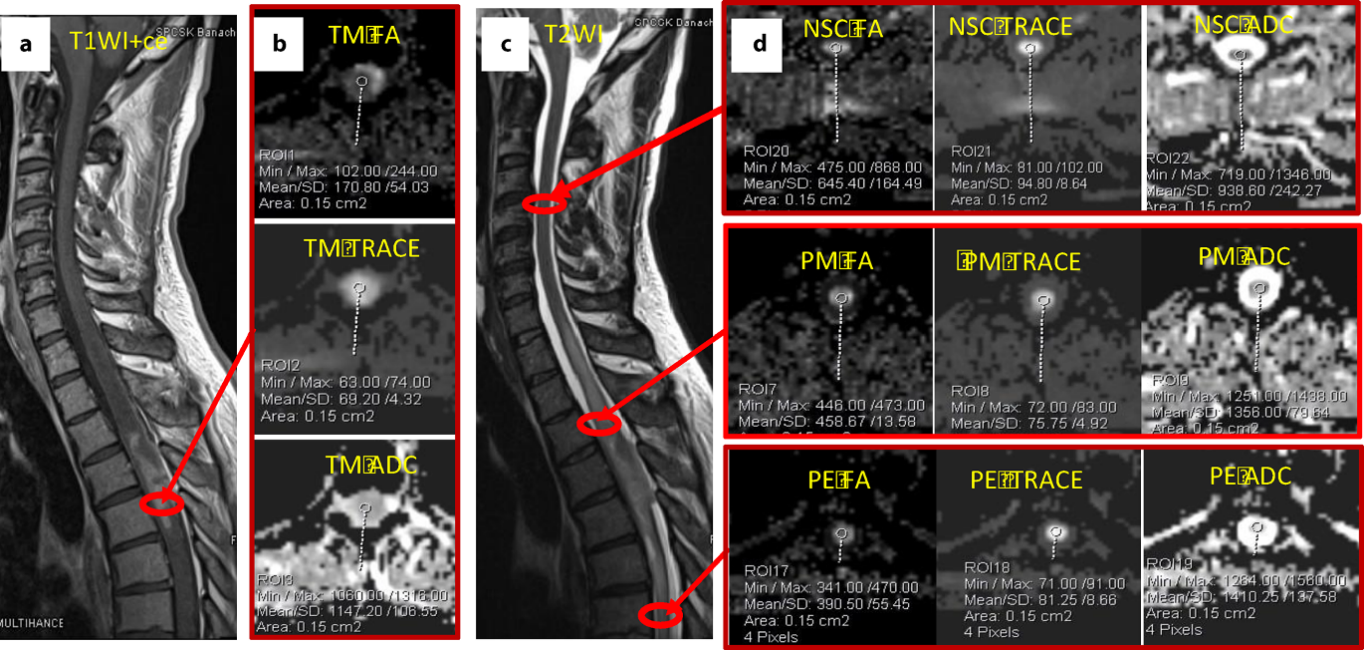
An example illustrating the method of collecting quantitative data. Astrocytoma grade II. Regions of interest (ROIs) placed within the enhancing tumor mass™ on contrast-enhanced T1-weighted image (**a**) with FA, TRACE, ADC measurements (**b**). ROIs placed within a peritumoral margin (*PM*), peritumoral edema (*PE*) and in normal appearing spinal cord (*NSC*) on T2-weighted image (**c**) with FA, TRACE, ADC measurements (**d**). *ADC* apparent diffusion coefficient, *FA* fractional anisotropy, *ROI* region of interest, *TRACE* trace of the diffusion tensor

In all cases, the DTI-derived metrics (FA, TRACE, ADC) were planned to be measured by placing the ROIs in four areas: in the contrast enhancing TM, in the T2-high signal area adjacent to the enhancing portion of the TM (defined as a peritumoral margin or immediate peritumoral region), in the T2-high signal area more distant from the TM (defined as peritumoral edema or distant peritumoral region), and in normal appearing spinal cord (Fig. 1). To better identify TM and tumor edema, the ROIs were placed in correlation with morphological T2W images and T1 gadolinium-enhanced images. At each location two measurements were made, for a total of eight ROIs. The DTI parameters FA, TRACE and ADC were calculated for each ROI so that 24 measurements should theoretically be obtained for each patient; however, the measurements were not feasible in all cases. Limitations included lack of visible peritumoral edema or the absence of normal appearing spinal cord in the examined area.

### Statistical Analysis

The results were compared between the two groups of tumors using nonparametric Mann–Whitney *U* test with statistical significance established at *p* < 0.05. Receiver operating characteristic (ROC) analysis was performed for each parameter (FA, TRACE, and ADC) on each level. Optimal thresholds for separating the IT and NIT were determined by maximizing the sum of specificity and sensitivity (i.e. the Youden index). The area under the ROC curve (AUC) was calculated.

Statistical analyses were performed using MedCalc Statistical Software (Ostend, Belgium) by one of the authors (WSz).

## Results

### Group Comparison

The group of ITs consisted of 3 astrocytomas, 2 glioblastomas, and 1 primitive neuroectodermal tumor. All NITs were ependymomas. In all patients with ITs and NITs, 2 measurements were obtained from a solid, contrast-enhancing part of the tumor, yielding a total of 12 measurements from ITs and 24 measurements from NITs.In all 6 patients with ITs, 2 measurements were obtained from each of 3 regions: the margin, edema, and the normal appearing spinal cord resulting in 17 measurements from the margin and the edematous region (7 measurements are missing due to the lack of a high T2 signal around the TM) in the group of patients with NITs, and 20 measurements from normal appearing spinal cord (4 measurements were not available due to the absence of regular spinal cord tissue in the examined area in some patients).

The mean value of the FA parameter for the TM in the NIT group was 0.205 (SD = 0.1), whereas in the IT group it was 0.228 (SD = 0.1, *p* < 0. 470). For margin, the mean FA value in NIT group was higher; specifically, 0.399 (SD = 0.08), compared to 0.304 in the IT (SD = 0.1), which gives a statistically significant value of *p* < 0.007. For edema, the mean FA value of NIT was higher: 0.439 (SD = 0.11) compared to the IT result of 0.350 (SD = 0.1), which gives a statistically significant value of *p* < 0.029. Statistical significance was not found for normal-appearing spinal cord (*p* < 0.087), and the mean FA value for the NIT was 0.617 (SD = 0.15), while it was 0.594 (SD = 0.07) for IT.

For the TRACE parameter, statistical significance was found in TM (*p* < 0.003), in margin (*p* < 0.017) and in edema (*p* < 0.016). The mean TRACE value in the TM in NIT was 0.091 and 0.135 in IT with the same standard deviation (SD = 0.04). The mean TRACE value in the margin in NIT was 0.086 (SD = 0.03), while it was 0.119 (SD = 0.04) in IT. Similar values were obtained in edema: 0.085 (SD = 0.03) and 0.110 (SD = 0.03), respectively. There was no significant difference for the TRACE measurements in normal appearing spinal cord: the mean value in NIT was 0.085 (SD = 0.03) and 0.103 (SD = 0.03) in IT, *p* < 0.102.

The ADC values obtained from the TM can enable differentiation with statistical significance between NIT and IT (*p* < 0.016). The mean ADC value in the TM in NIT was 1.59 (SD = 0.55), and 1.12 (SD = 0.2) in IT. There was no statistical significance of the ADC parameter for margin, edema and normal appearing spinal cord; however, *p* value of the ADC in edema is on the border of the set significance level (*p* < 0.054). The mean ADC value in edema for NIT was 1.36 (SD = 0.22), and 1.17 (SD = 0.21) for IT. The margin’s mean ADC in NIT was 1.34 (SD = 0.28), and in IT 1.18 (SD = 0.16), which gives *p* < 0.259. In normal appearing spinal cord in patients with NIT, the mean ADC value was 0.99 (SD = 0.11), while it was 1.01 (SD = 0.12) in IT patients with *p* < 0.846.

In summary, statistical analysis using the nonparametric Mann-Whitney *U* test, showed that the strongest parameter differentiating the group of patients with NIT and IT was TRACE, whose value showed statistical differences within tumor mass (*p* < 0.003), margin (*p* < 0.017), and edema (*p* < 0.016). For the FA parameter, statistical differences were found within the peritumoral margin (*p* < 0.007) and edema (*p* < 0.029), and for the ADC parameter only within tumor mass (*p* < 0.016). None of the parameters showed a statistical difference in normal appearing spinal cord: FA (*p* < 0.087), TRACE (*p* < 0.102), ADC (*p* < 0.846) (Table 2).

**Table 2:** Average results for functional anisotropy (FA), diffusivity (TRACE), and apparent diffusion coefficient (ADC) parameters in patients with infiltrating (IT) and non-infiltrating (NIT) spinal cord tumors, including statistical differences (nonparametric Mann-Whitney *U* test)

	*N* – NIT	*N* – IT	FA			TRACE			ADC		
			Mean results – NIT	Mean results – IT	*p*-value	Mean results – NIT	Mean results – IT	*p*-value	Mean results – NIT	Mean results – IT	*p*-value
Tumor mass	24	12	0.205 ± 0.1	0.228 ± 0.1	0.470	*0.091* *±* *0.04*	*0.135* *±* *0.04*	**0.003**	*1.59* *±* *0.55*	*1.12* *±* *0.2*	**0.016**
Peritumoral margin	*17*	*12*	*0.399* *±* *0.08*	*0.304* *±* *0.1*	**0.007**	*0.086* *±* *0.03*	*0.119* *±* *0.04*	**0.017**	1.34 ± 0.28	1.18 ± 0.16	0.259
Peritumoral edema	*17*	*12*	*0.439* *±* *0.11*	*0.350* *±* *0.1*	**0.029**	*0.085* *±* *0.03*	*0.110* *±* *0.03*	**0.016**	*1.36* *±* *0.22*	*1.17* *±* *0.21*	***0.054***
Normal spinal cord	20	12	0.617 ± 0.15	0.594 ± 0.07	0.087	0.085 ± 0.03	0.103 ± 0.03	0.102	0.99 ± 0.11	1.01 ± 0.12	0.846

*ADC* apparent diffusion coefficient, *FA* functional anisotropy, *IT* infiltrating tumors, *NIT* noninfiltrating tumors, *N *– IT number of measurements of the infiltrating tumors, *N* – NIT number of measurements of the non-infiltrating tumors, *TRACE* trace of the diffusion tensor

Statistical significance established at *p* < 0.05

### ROC Analysis

**Fig. 2:**
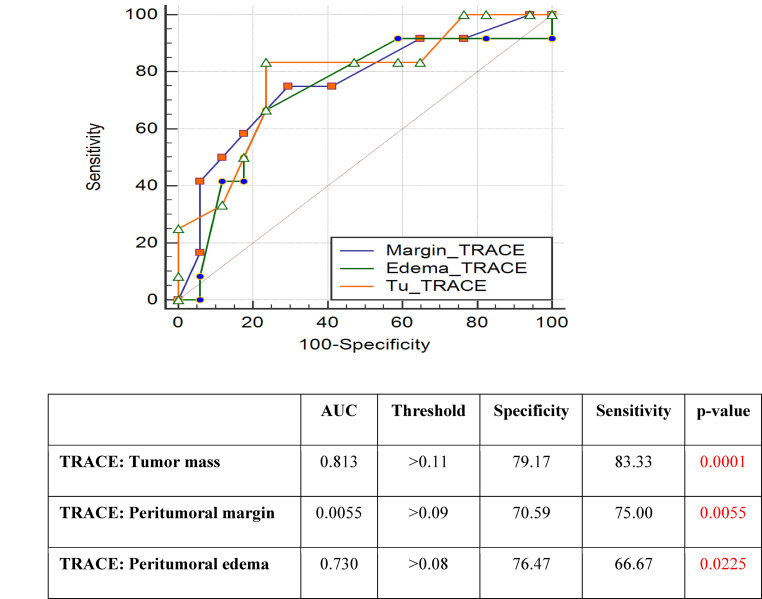
ROC curve analysis for TRACE parameter. Significant differences in tumor mass (*p* < 0.0001), peritumoral margin (*p* < 0.0055), and peritumoral edema measurements (*p* < 0.0225) were found. High specificity and sensitivity in the differentiation of NIT and IT were shown for the TRACE parameter: 79.17% and 83.33% for tumor mass, 70.59% and 75% for peritumoral margin, and 76.47% and 66.67% for peritumoral edema. *AUC* area under the ROC curve, *IT* infiltrating tumor, *NIT* non-infiltrating tumor, *ROC* receiver operating characteristic, *Specificity and Sensitivity*—optimal thresholds for separating the ITs and NITs, *Threshold*—optimal thresholds for maximizing the sum of specificity and sensitivity calculated for each parameter and each location, *TRACE*—trace of the diffusion tensor

Similar results were obtained in the ROC curve analysis. Statistical significance was demonstrated for the TRACE parameter with respect to tumor, margin and edema (*p* < 0.0001, *p* < 0.0055, *p* < 0.0225, respectively). This parameter also showed the highest specificity and sensitivity in the differentiation of NIT and IT (79% and 83% for tumor, 71% and 75% for margin, and 76% and 67% for edema). The ROC curves and areas under the ROC curve for TRACE are shown in Fig. 2.

**Fig. 3:**
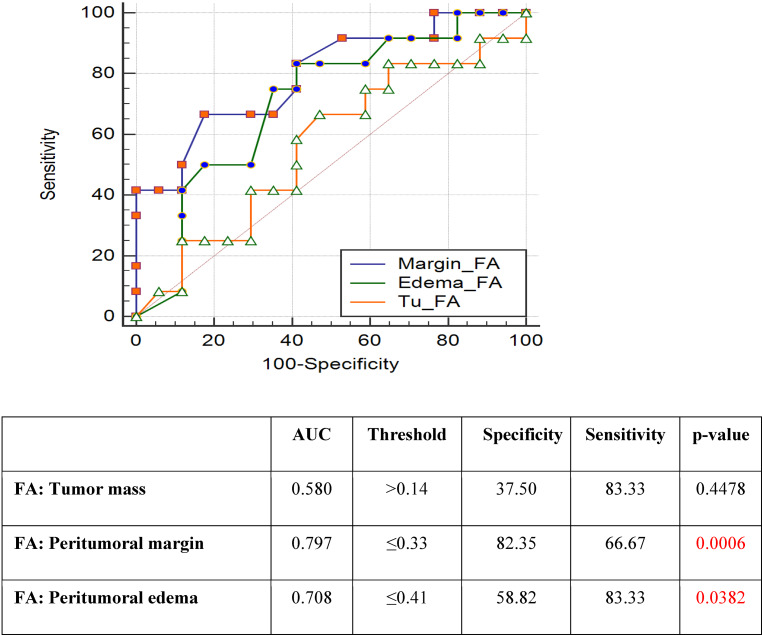
ROC curve analysis for FA parameter. Peritumoral margin and peritumoral edema measurements achieved statistical significance with *p* < 0.0006 and *p* < 0.0382, respectively. The highest specificity and sensitivity in differentiation between the NIT and the IT was determined for peritumoral margin parameter: 82.35% and 66.67%. *AUC* area under the ROC curve, *FA* fractional anisotropy, *IT* infiltrating tumor, *NIT* non-infiltrating tumor, *ROC* receiver operating characteristic, *Specificity and Sensitivity*—optimal thresholds for separating the ITs and NITs, *Threshold*—optimal thresholds for maximizing the sum of specificity and sensitivity calculated for each parameter and each location

For the FA parameter, statistical significance was found in margin (*p* < 0.0006) and edema (*p* < 0.0382) measurements, and for the ADC parameter in tumor (*p* < 0.0018) and edema (*p* < 0.0396) measurements. The specificity and sensitivity of the FA parameter in differentiating between the NIT and the IT were the highest in the margin and represented 82% and 67%, respectively, 59% and 83% in edema, and 37% and 83% in tumor. The ROC curves and areas under the ROC curve for FA are shown in Fig. 3.

**Fig. 4:**
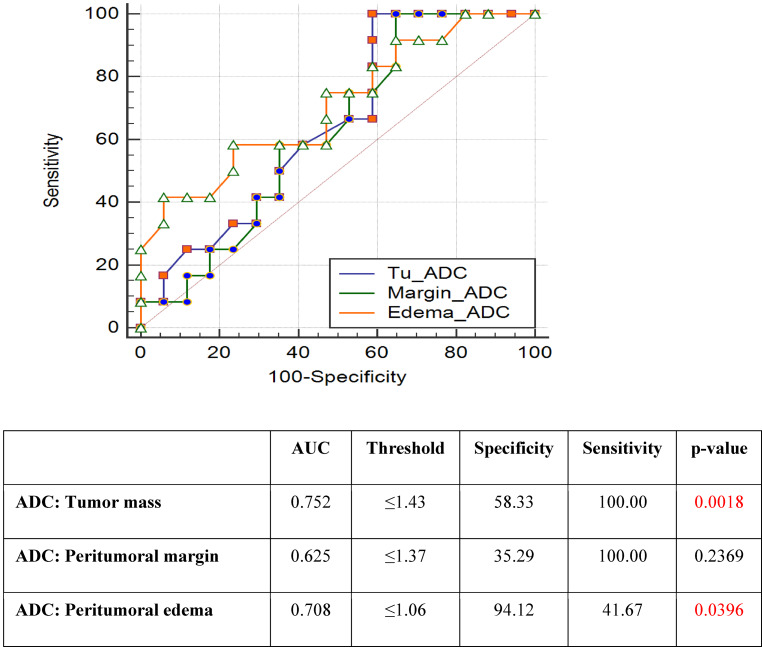
ROC curve analysis for ADC parameter. Differentiation between NIT and IT was performed with 100% sensitivity, but lower specificity: 58.33% for tumor mass and 35.29% for peritumoral margin. ADC parameter revealed statistical significance *p* < 0.0018 for tumor mass and *p* < 0.0396 for peritumoral edema measurements. *ADC* apparent diffusion coefficient, *AUC* area under the ROC curve, *IT* infiltrating tumor, *NIT* non-infiltrating tumor, *ROC* receiver operating characteristic, *Specificity and Sensitivity*—optimal thresholds for separating the ITs and NITs, *Threshold*—optimal thresholds for maximizing the sum of specificity and sensitivity calculated for each parameter and each location

The ADC parameter allowed a differentiation between NIT and IT with 100% sensitivity in the region of tumor and margin, while the specificity was 58% and 34%, respectively. In terms of edema, sensitivity was only 42%, while the specificity was 94%. ROC curves and areas under the ROC curves and areas under the ROC for ADC are shown in Fig. 4. For each parameter and each location, optimal thresholds for maximizing the sum of specificity and sensitivity were also calculated (Figs. 2, 3 and 4).

## Discussion

The DTI technique has been widely used in recent scientific research for the differentiation of infiltrating and noninfiltrating intracranial neoplasms. The method is most often applied to differentiate primary brain tumors such as glioblastoma with infiltrative patterns of growth from noninfiltrating metastatic masses. It also used to discriminate between glial tumors of various malignancy and resultant various degrees of invasion of surrounding tissue. These studies were based on the assumption that actual tumor margins can extend microscopically for several centimeters beyond the radiographically detected (contrast-enhanced) margin of disease. Both ITs and NITs are surrounded by extensive areas of T2-hyperintensity, recognized to be vasogenic edema. In NITs, this peritumoral zone is thought to consist of pure water, while in ITs such as glioblastoma, this peritumoral zone has often been shown to contain tumor cells that have spread into the edematous tissue along the white matter tracts [11]. This difference in the peritumoral zone cellularity affects DTI parameters. A similar hypothesis can be assumed for infiltrating and non-infiltrating spinal cord tumors. This analysis showed that the statistically significant differences between IT and NIT in the DTI metrics alteration are evident in TM, peritumoral margin and edema, most probably reflecting biological properties of the tumor tissue. The TRACE proved to be the most important differentiating parameter. The fact that none of the parameters showed a statistical difference in normal appearing spinal cord suggests that this research method is viable. The mean FA value in normal appearing spinal cord was 0.617 ± 0.15 for NIT and 0.594 ± 0.07 for IT, which is in line with other studies [12] and is comparable to white matter tracts in the brain.

This paper focused specifically on the peritumoral zone; however, statistical differences between IT and NIT in TRACE and ADC value were also found in the tumor mass itself. Within the material glial tumors of different malignancy (WHO grade II, III and IV) were found among IT, whereas in NIT all tumors were higher differentiated ependymomas (WHO grade II). Lower ADC value in IT (1.12 ± 0.20) compared to NIT (1.59 ± 0.55) is in keeping with the findings in high grade brain tumors and indicates higher cellularity [13, 14].

The results of the systematic review and meta-analysis of the DTI values in brain tumor differentiation published by Jiang et al. [15] are ambiguous. Compared to contralateral normal appearing white matter, FA values in lesions of gliomas or metastases have been reported to be consistently reduced [16–18]. In contrast, some authors reported significantly higher FA from the peritumoral region of high-grade gliomas as compared to brain metastases [19, 20]; however, some studies drew utterly contrary conclusions [16, 18, 21]. Similar controversy exists regarding the value of mean diffusivity for discerning gliomas from brain metastases [16, 20].

In recent years, many efforts have been undertaken to apply the DTI technique in several spinal cord pathologies [22–26], and only a few have focused on intramedullary spinal cord tumors [27–29]. Ducreux et al. [27] were the first to calculate the FA value in the solid part of five intramedullary astrocytomas. All patients had decreased FA values in the mass tumor site (FA 0.48 ± 0.02) compared to the spinal cord of healthy volunteers (FA 0.74 ± 0.04). In the present study, the mean FA result of TM was much lower in IT (0.228 ± 0.1), as well as in NIT (0.205 ± 0.1), and only slightly lower in normal-appearing spinal cord (0.594 ± 0.07 for IT and 0.617 ± 0.15 for NIT).

Zhao et al. [29] analyzed 11 patients with clinically diagnosed cervical spinal cord astrocytomas, along with 10 healthy volunteers. The mean FA value of the lesions (within the mass and excluding cysts) was 0.24 ± 0.11 in the 11 patients, similar to our IT group (0.228 ± 0.1). The mean ADC value of the lesions in the 11 patients was 1.50 ± 0.52 × 10^−3^ mm^2^/s, whereas it was 1.12 ± 0.2 in our IT group. The paper referred to was deficient in that astrocytomas were confirmed by surgery in only 3 of the 11 patients enrolled in the study, while the others were clinically followed for 6–24 months.

In 2013 Setzer et al. [28] assessed the integrity of white matter tracts based on diffusion tensor tractography, without quantitative analysis of DTI parameters. Based on fiber content in the TM, they grouped 13 spinal cord tumors into 3 categories, and then translated this classification into resectable and nonresectable. Using these categories, 6 ependymomas were classified as resectable, 2 ependymomas, 2 astrocytomas, 2 lymphomas and 1 multiple sclerosis plaque were classified as nonresectable. In this small study, a significant statistical association could not be established; however, it was observed that the NITs were unlikely to penetrate fibers and were as a result often rated as resectable.

Although the groups of patients in this study were more homogeneous and larger than in the previous studies, the analysis still has the limitations of a small number of patients enrolled, and technical difficulties which restrict the use of the DTI method in the lower thoracic and lumbar spine. Further studies with a larger number of patients are needed to corroborate these preliminary results.

## Conclusion

The DTI technique, previously utilized for the imaging of brain tumors, can also improve the preoperative differentiation between infiltrating and non-infiltrating intramedullary spinal cord tumors. Quantitative analysis of DTI parameters of spinal cord tissue surroundings spinal masses may be useful for the distinction between these two different types of tumors and is an important requirement for surgical planning and successful treatment outcomes.

## Abbreviations

ADC: Apparent diffusion coefficient
AUC: Area under the curve
DTI: Diffusion tensor imaging
FA: Fractional anisotropy
IT: Infiltrating tumor
NIT: Noninfiltrating tumor
ROC: Receiver operating characteristic
ROI: Region of interest
TM: Tumor mass
TRACE: Trace of the diffusion tensor
